# The blockade of the transient receptor potential vanilloid type 1 and fatty acid amide hydrolase decreases symptoms and central sequelae in the medial prefrontal cortex of neuropathic rats

**DOI:** 10.1186/1744-8069-7-7

**Published:** 2011-01-17

**Authors:** Vito de Novellis, Daniela Vita, Luisa Gatta, Livio Luongo, Giulia Bellini, Maria De Chiaro, Ida Marabese, Dario Siniscalco, Serena Boccella, Fabiana Piscitelli, Vincenzo Di Marzo, Enza Palazzo, Francesco Rossi, Sabatino Maione

**Affiliations:** 1 Department of Experimental Medicine, Division of Pharmacology, Second University of Naples, via Costantinopoli 16, 80138 Naples, Italy; 2 Institute of Biomolecular Chemistry, Consiglio Nazionale delle Ricerche, Via Campi Flegrei 34, Pozzuoli (NA), Italy

## Abstract

**Background:**

Neuropathic pain is a chronic disease resulting from dysfunction within the "pain matrix". The basolateral amygdala (BLA) can modulate cortical functions and interactions between this structure and the medial prefrontal cortex (mPFC) are important for integrating emotionally salient information. In this study, we have investigated the involvement of the transient receptor potential vanilloid type 1 (TRPV1) and the catabolic enzyme fatty acid amide hydrolase (FAAH) in the morphofunctional changes occurring in the pre-limbic/infra-limbic (PL/IL) cortex in neuropathic rats.

**Results:**

The effect of *N*-arachidonoyl-serotonin (AA-5-HT), a hybrid FAAH inhibitor and TPRV1 channel antagonist, was tested on nociceptive behaviour associated with neuropathic pain as well as on some phenotypic changes occurring on PL/IL cortex pyramidal neurons. Those neurons were identified as belonging to the BLA-mPFC pathway by electrical stimulation of the BLA followed by hind-paw pressoceptive stimulus application. Changes in their spontaneous and evoked activity were studied in sham or spared nerve injury (SNI) rats before or after repeated treatment with AA-5-HT. Consistently with the SNI-induced changes in PL/IL cortex neurons which underwent profound phenotypic reorganization, suggesting a profound imbalance between excitatory and inhibitory responses in the mPFC neurons, we found an increase in extracellular glutamate levels, as well as the up-regulation of FAAH and TRPV1 in the PL/IL cortex of SNI rats. Daily treatment with AA-5-HT restored cortical neuronal activity, normalizing the electrophysiological changes associated with the peripheral injury of the sciatic nerve. Finally, a single acute intra-PL/IL cortex microinjection of AA-5-HT transiently decreased allodynia more effectively than URB597 or I-RTX, a selective FAAH inhibitor or a TRPV1 blocker, respectively.

**Conclusion:**

These data suggest a possible involvement of endovanilloids in the cortical plastic changes associated with peripheral nerve injury and indicate that therapies able to normalize endovanilloid transmission may prove useful in ameliorating the symptoms and central sequelae associated with neuropathic pain.

## Introduction

There is increasing evidence that the unpleasantness or affective component of pain, similarly to other high-order cognitive and emotional functions (i.e. decision making, goal-directed behavior, and working memory) [1,2], are driven by specific forebrain areas, and, among these, **the **prefrontal cortex (PFC) plays a pivotal role. In particular, the medial prefrontal cortex (mPFC) participates in signalling the unpleasantness of pain in humans [3,4], being the affective component of pain under the control of the anterior cingulate cortex [5,6]. Supraspinal brain regions are profoundly affected by peripheral nerve injury or spinal nerve transection in rodents [7,8]. Accordingly, patients with chronic back pain showed cortex morpho-functional frontal atrophy [9]. Neural reorganization of the mPFC might occur and account for the impaired performance of emotional decision making tasks (i.e. the Iowa Gambling Task) [10] in patients suffering from complex region pain syndrome type I (CRPS I) or chronic back pain similarly to patients with frontal cortex lesions. The extent of activation of the mPFC during spontaneous pain and the extent of emotional and cognitive impairment correlates to the intensity and the duration of the pain condition in patients suffering from chronic back pain [11]. Human brain imaging studies have thus revealed that chronic pain is associated with the activation of excitatory and inhibitory neurotransmission, neurotrophic factor transcription and synthesis of proteins involved in glutamate receptor expression, along with GABAergic neuron apoptosis and new cortical connection establishment [12]. Enhanced pain perception [13-15] has been shown to be associated with over-expression of the NR2B subunit of the NMDA receptor and morphological reorganization in the anterior cingulate cortex [10]. Larger NMDA-mediated currents were also observed in pyramidal cells of the infralimbic cortex in neuropathic rats, corresponding to the mPFC of primates [16]. Moreover, in a more recent study, local application of D-cycloserine, an NMDA partial agonist, generated an anti-allodynic effect closely correlated with the infusion site in a way that the maximum effect was observed in the prelimbic (PL) cortex. Chronic pain can clearly interfere with the mPFC which plays a critical role in the neurophysiological processes such as a reorganization of synaptic and neural functioning [17,18], which in turn, could be responsible for the impaired effectiveness of emotional decision making test.

The basolateral amygdala (BLA) can modulate cortical functions, and interactions between the BLA and mPFC are important for integrating emotionally salient information [19-24]; indeed the activation of BLA can modulate the activity of separate subpopolations of mPFC neurons [25-28]. Recent works have shown that pain-related plasticity in the central nucleus of the amigdala (CeA) contributes critically to the emotional affective component of pain [29-34]. Among the novel targets identified for chronic pain therapy, the transient receptor potential vanilloid subtype 1 (TRPV1) is attracting increasing interest, since it plays a central role in the transduction of pain and the initiation of the neurogenic inflammatory responses including cancer pain [35-38]. The expression and sensitivity of TRPV1 are enhanced during inflammation and neuropathic pain leading to a lowering of the pain threshold [39]. Apart from peripheral sensory neurons [36], TRPV1 is also expressed in the brain [40-44], including those areas involved in pain processing, such as the periaqueductal grey (PAG) and cingulate cortex [45,46]. TRPV1 has been shown to be physiologically active in some nuclei of the central nervous system [47,48]. Based on recent evidence that *N*-arachidonoyl-serotonin (AA-5-HT, a unique compound with the "dual" ability to inhibit fatty acid amide hydrolase [FAAH], the catabolic enzyme of endocannabinoids/endovanilloids, and antagonize TRPV1), shows analgesic activity in acute or chronic pain models in rodents [49,50], in this study we have investigated the effect of repeated systemic administration of AA-5-HT on: i) inhibitory and excitatory activity of the perilimbic/infra-limbic (PL/IL) cortex neurons, be it spontaneous, or evoked by electrical stimulation of the BLA, or by mechanical stimulation of the hind paw; ii) extracellular glutamate and GABA levels in PL/IL cortex in awake rats; and iii) phenotypic changes of inhibitory and excitatory PL/IL cortex neurons in SNI rats. Moreover, we assessed FAAH and TRPV1 expression and endovanilloid levels in the PL/IL cortex of sham and neuropathic rats, and the mechanical allodynia associated with neuropathic pain after a single intra mPFC administration of vehicle or AA-5-HT.

## Materials and methods

### Animals and surgery

Male Wistar rats (220-250 g) were housed 3 per cage under controlled illumination (12:12 h light:dark cycle; light on 06.00 h) and standard environmental conditions (ambient temperature 20-22°C, humidity 55-60%) for at least 1 week before the commencement of experiments. Rat chow and tap water were available *ad libitum*. The experimental procedures were approved by the Animal Ethics Commitee of the Second University of Naples. Animal care was in compliance with Italian (D.L. 116/92) and EEC (O.J. of E.C. L358/1 18/12/86) regulations on the protection of laboratory animals. All efforts were made to minimise animal suffering and to reduce the number of animals used.

Mononeuropathy was induced through spinal nerve ligation (SNI) according to the method of Decostered and Woolf [51]. Rats were anaesthetized with sodium pentobarbital (50 mg/kg i.p.). The sciatic nerve was exposed at mid-thigh level distal to the trifurcation and freed of connective tissue; the three peripheral branches (sural, common peroneal, and tibial nerves) of the sciatic nerve were exposed without stretching nerve structures. Both tibial and common peroneal nerves were ligated and transected together. The sham procedure consisted of the same surgery without ligation and transection of the nerves.

### Treatments

For in vivo extracellular recording experiments groups (n = 10) of sham and SNI rats were treated for 7 days with vehicle (0.5% DMSO in ACSF) or AA-5-HT (5 mg/kg i.p.). Groups (n = 8-10) of sham and SNI rats were used for the assessment of mechanical allodynia 7 days after surgery before and after a single intra-cortex microinjections of vehicle (0.5% DMSO in ACSF), AA-5-HT (0.1-0.25-1 nmol), URB597 (1-2-4 nmol), I-RTX (0.25-0.5-1 nmol) or AM251 (0.25-0.5 nmol). Moreover, additional groups of sham and SNI rats treated with vehicle and following behavioural tests for ascertaining the occurrence of allodynia in SNI rats, were divided into three further groups (n = 3) for RT-PCR, western blot and immunohistochemistry. Finally, for the microdialysis experiments sham (n = 7) and SNI rats (n = 8) have been tested 7 days after surgery.

### Nociceptive behaviour (allodynia)

Mechanical allodynia was measured by using Dynamic Plantar Aesthesiometer (Ugo Basile, Varese, Italy). Rats were allowed to move freely in one of the two compartments of the enclosure positioned on the metal mesh surface. Rats were adapted to the testing environment before any measurement was taken. The mechanical stimulus was then delivered to the plantar surface of the hind paw of the rat from below the floor of the test chamber by an automated testing device. A steel rod (2 mm) was pushed with ascending force (0-30 g in 10 sec). When the animal withdrew its hind paw, the mechanical stimulus was automatically withdrawn and the force recorded to the nearest 0.1 g. Nociceptive responses for mechanical sensitivity were expressed as mechanical withdrawal threshold (MWT) in grams.

Sham and SNI rats received a single administration of vehicle, AA-5-HT (0.1-0.25-1 nmol), URB597 (1-2-4 nmol), I-RTX (0.25-0.5-1 nmol) or **AM251 (0.25-0.5 nmol) **into the PL/IL cortex 7 day after the sciatic nerve insult. The AA-5-HT dose was chosen based on our previous study in which it proved to be effective in several pain models in rodents [49]. Each rat served as its own control, the responses being measured both before and after vehicle or drug administration. MWT was quantified by an observer who was blind to the treatment.

### In vivo single unit extracellular recording

**Figure 1:**
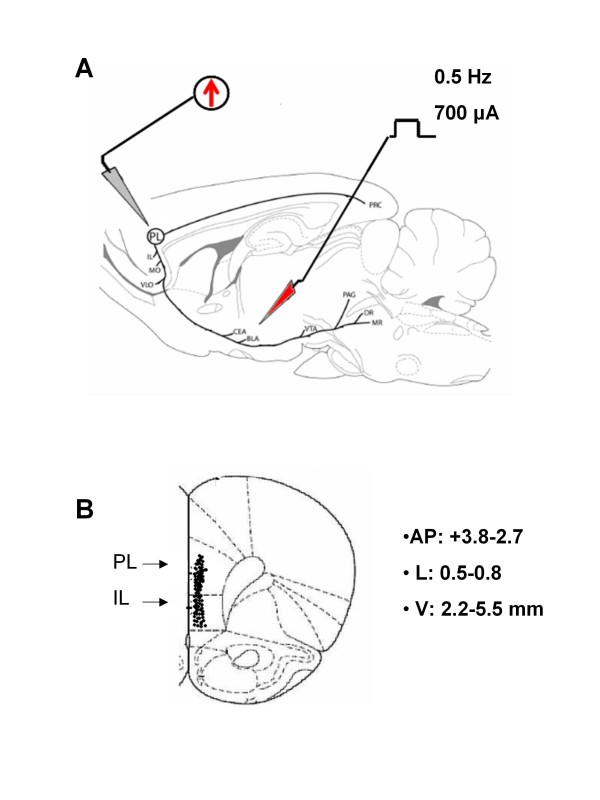
**Representative schematic illustration showing the location of the recording electrode in the PL/IL cortex and of the stimulation electrode in the BLA (A)**. Number values refer to stimulation parameters. Abbreviations: CEA, central nucleus of amygdala; BLA, basolateral nucleus of amygdala; VTA, ventral tegmental area; PAG, periaqueductal gray; DR, dorsal raphe nucleus; PL, prelimbic cortex; IL, infralimbic cortex; MO, medial orbital cortex; PRC, perirhinal cortex. Reprinted from Vertes [99]. Schematic illustration of the PL/IL cortex recording sites (B). Filled circles represent mPFC cell recorded sites. Number values indicate distance to bregma.

Rats for electrophysiological recordings were anaesthetised with pentobarbital (50 mg/kg, i.p.) and placed in a stereotaxic device (David Kopf Instruments, Tujunga, CA, USA). Body temperature was maintained at 37°C with a temperature-controlled heating pad. In all surgical preparations, the scalp was incised and holes were drilled in the skull overlying the site of recording, mPFC (AP: +3.8-2.7, L: 0.5-0.8 and V: 2.2-5.5 mm), and the site of stimulation, BLA (AP: -2.5 -3.1, L: 4.5-5.0 and V: 7.2-9) (Figure 1) according to the coordinates from Paxinos and Watson [52] and contralateral with respect to the nerve insult. Anaesthesia was maintained with a constant, continuous infusion of propofol (5-10 mg/kg/h, i.v.) and a bipolar concentric electrode (NEX-100; Rhodes Medical Instruments Inc., Summerland, CA) connected to A320 stimulator (World Precision Instruments England) was lowered into the caudal region of the BLA according to Floresco and Tse [53]. After lowering the stimulating electrode into the BLA (Figure 1A), a glass-insulated tungsten filament electrode (3-5 MΩ) (FHC Frederick Haer & Co., ME, USA) was stereotaxically lowered into the mPFC (Figure 1B). The recorded signals were amplified and displayed on a digital storage oscilloscope to ensure that the unit under study was unambiguously discriminated throughout the experiment. Signals were also fed into a window discriminator, whose output was processed by an interface CED 1401 (Cambridge Electronic Design Ltd., UK) connected to a Pentium III PC. Spike2 software (CED, version 4) was used to create peristimulus rate histograms on-line and to store and analyse digital records of single unit activity off-line. Configuration, shape, and height of the recorded action potentials were monitored and recorded continuously using a window discriminator and Spike2 software for on-line and off-line analysis.

This study only included neurons with a regular spiking pattern and a spontaneous firing rate between 0.4 and 1.5 Hz, which were classified as pyramidal neurons [53-55]. Once a neuron was encountered in the mPFC, the position of the microelectrode was adjusted to maximize the spike amplitude relative to background noise. We then delivered electrical stimuli into the BLA (700 μA) at 2 sec intervals. At least 50 single pulses were delivered to generate peristimulus time histograms (PSTHs). Once the cell was identified, mechanical stimuli were applied to the hind paw (contralateral to the mPFC) by using a home made spring-operated forceps that closed with a force (>500 and <2000 g/10 mm^2^) calibrated with a tension spring balance and delivered for 5 sec [56]. By using electrical (BLA) or mechanical (hind paw) stimuli we were able to determine whether each individual neuron was inhibited, excited or showed no response to stimulation. We did not record data for neurons that displayed no change in firing as a result of stimulation and continued the cell-searching procedure.

### Characterization of BLA-evoked responses and stimulation protocol

We observed that BLA stimulation could evoke two distinct types of firing changes in separate populations of mPFC responding neurons. The more commonly observed response was a robust inhibition of neural activity. We characterized these responses accordingly with previously established criteria used by Ishikawa and Nakamura [26]. Specifically, a cell was considered to be inhibited by BLA stimulation if it displayed a complete cessation of spontaneous firing after BLA stimulation. Neurons displaying this type of response are referred to hereafter as BLA→mPFC(-) neurons [53]. Only neurons that displayed a spontaneous firing rate of between 0.5 and 1.5 Hz were used for the data analysis.

**Figure 2:**
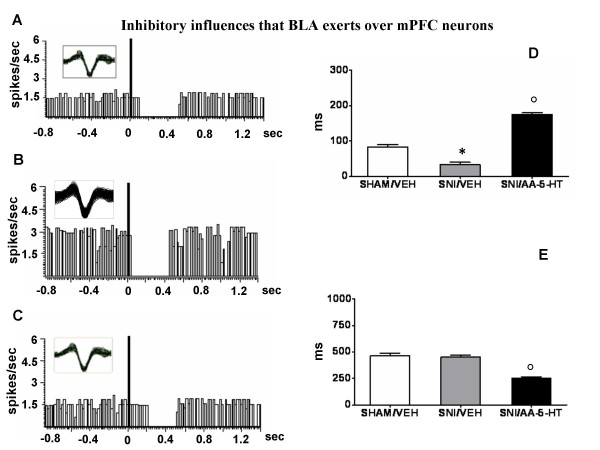
**BLA stimulation evokes inhibitory responses in the PL/IL cortex of BLA → PL/IL (-) neurons**. The figure shows different parameters of BLA-evoked inhibition, including the onset and duration of the inhibition, in sham and SNI rats treated for 7 days with vehicle (veh) or AA-5-HT (5 mg/kg, i.p.). "A", "B" and "C" show representative ratemater records of a BLA → PL/IL (-) neurons of sham/veh, SNI/veh or SNI/AA-5-HT rat, respectively. "D" and "E" show the onset and duration (mean ± SEM) of the inhibition, respectively, in different groups of rats. * indicates statistically significant difference versus sham/veh and º versus SNI/veh. P < 0.05 was considered as value of significance and n = 10 was used for each group.

Once a neuron that was inhibited by BLA stimulation was isolated, single-pulse stimulation was delivered at 0.5 Hz. We typically used 100-250 sweeps and peristimulus time histograms were generated on-line. We used two parameters derived from the peristimulus time histograms to assess differences between different groups of rats. Our primary measure was the "duration" of inhibition (ms) as defined by Ishikawa and Nakamura [26]. The duration was calculated from the longest period when spontaneous firing was completely suppressed after BLA stimulation. The second measure we have considered here was the "onset" of this period of inhibition (ms) after BLA stimulation (the time interval between the stimulus application and last spike before a complete cessation of neuronal activity). By using these parameters we could have a reliable index of BLA-evoked inhibition and changes in the inhibitory influences that BLA inputs exert over the mPFC neuron firing. An example of a typical inhibitory response recorded from a BLA → mPFC(-) neuron is shown in Figure 2A

**Figure 3:**
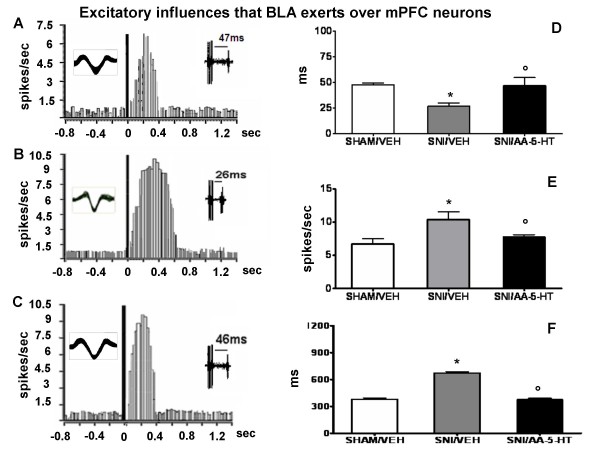
**BLA stimulation evokes excitatory responses in the PL/IL cortex of BLA → PL/IL (+) neurons**. The figure shows different parameters of BLA-evoked excitation, including the onset, the frequency and the duration of excitation, in sham and SNI rats treated for 7 days with vehicle (veh) or AA-5-HT (5 mg/kg, i.p.). "A", "B" and "C" show a representative ratemater record of a BLA → PL/IL (+) neurons of sham/veh, SNI/veh and SNI/AA-5-HT rat. "D","E" and F show the onset, onset, the frequency and the duration (mean ± SEM) of the excitation, respectively, in the different groups of rats. * indicates statistically significant difference versus sham/veh and º versus SNI/veh. P < 0.05 was considered as value of significance and n = 10 was used for each group.

A second group of neurons displaying a fast-onset burst of firing were classified as BLA → mPFC(+) neurons. This group of neurons showed a cluster of spikes typically showing a Gaussian pattern of distribution and appeared to have an increased probability of spike firing after BLA stimulation (700 μA). From the peristimulus time histograms we measured the "duration" of excitation (in ms) as the period of the increased firing activity which exceeds the average baseline value + 2 standard deviation (SD). Moreover, we measured the frequency of evoked excitation and the onset of excitation which was considered as the time from the application of the stimulus (artefact) to the first evoked spike which exceeds the average baseline value + 2 SD. The onset of burst (ms) was calculated as the time interval between the stimulus application and the first evoked spike of the burst [22,57]. These criteria were used as an index of changes in the excitatory influence that BLA inputs exert over mPFC neuron firing. An example of a typical excitatory response recorded from a BLA → mPFC(+) neuron is shown in Figure 3A.

### Characterization of mechanical-evoked responses

**Figure 4:**
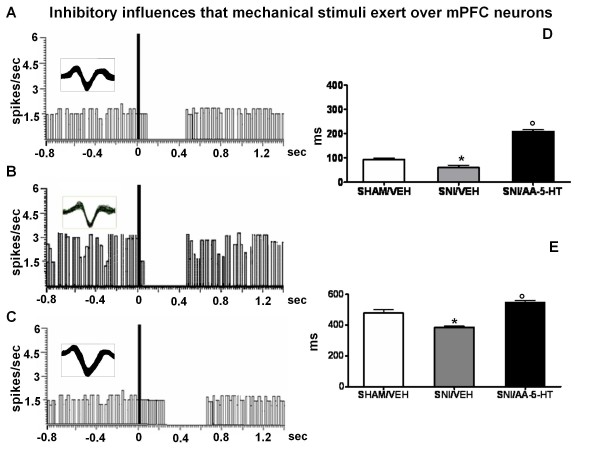
**Mechanical nociceptive stimulation evokes inhibitory responses in the PL/IL cortex of BLA → PL/IL (-) neurons**. The figure shows different parameters of mechanical nociceptive stimulation-evoked inhibition, including the onset and duration of the inhibition, in sham and SNI rats treated for 7 days with vehicle (veh) or AA-5-HT (5 mg/kg, i.p.). "A", "B" and "C" show a representative ratemater record of a mechanical stimulus → PL/IL (-) neurons of sham/veh, SNI/veh and SNI/AA-5-HT rat, respectively. "D" and "E" show the onset and duration (mean ± SEM) of the inhibition, respectively, in the different groups of rats. * indicates statistically significant difference versus sham/veh and º versus SNI/veh. P < 0.05 was considered as value of significance and n = 10 was used for each group.

**Figure 5:**
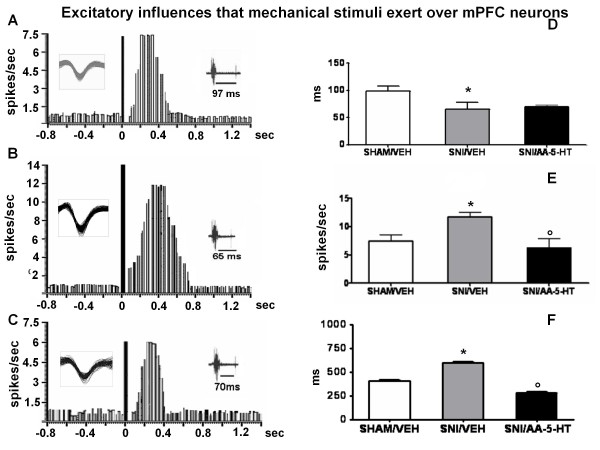
**Mechanical stimulation evokes excitatory responses in the PL/IL cortex neurons of BLA → PL/IL (+) neurons**. The figure shows different parameters of mechanical stimulus-evoked excitation, including the onset, the frequency and the duration of excitation, in sham and SNI rats treated for 7 days with vehicle (veh) or AA-5-HT (5 mg/kg, i.p.). "A", "B" and "C" show a representative ratemater record of a mechanical stimulus → PL/IL (+) neurons of sham/veh, SNI/veh and SNI/AA-5-HT rat. "D","E" and F show the onset, the frequency and the duration (mean ± SEM) of the excitation, respectively, in the different groups of rats. * indicates statistically significant difference versus sham/veh and º versus SNI/veh. P < 0.05 was considered as value of significance and n = 10 was used for each group.

Mechanical stimuli were applied to the hind paw (contralateral to the mPFC) by a spring-operated forceps with a force (>500 and <2000 g/10 mm^2^) which squeezed the tissue (painful pressure). The stimulus duration was 5 sec. The mechanical stimulus evoked inhibitory or excitatory response in separate populations of mPFC neurons. The same parameters of the inhibitory and excitatory responses were measured and considered as an index of mPFC neuron firing response to mechanical noxious stimuli. An example of a typical inhibitory and excitatory response recorded from a mechanical stimulation on mPFC (-) and mPFC (+)neurons is shown in Figure 4A and 5A respectively.

### In vivo microdialysis

Brain microdialysis experiments were performed in awake and freely moving rats. In brief, rats were anaesthetised with pentobarbital (50 mg/kg, i.p.) and stereotaxically implanted with concentric microdialysis probes into the mPFC using coordinates: AP: +3.8-2.7 mm, L: 0.4-0.7 mm from bregma and V: 5.3 mm below the dura. Microdialysis concentric probes were constructed as described by Hutson et al., [58] with 25 G (0.3 mm I.D., 0.5 mm O.D.) stainless steel tubing: inlet and outlet cannulae (0.04 mm I.D., 0.14 mm O.D.) consisted of fused silica tubing (Scientific Glass Engineering, Melbourne, Australia). The microdialysis probe had a tubular active membrane (Enka AG, Wuppertal, Germany) of 3 mm in length. Following a post-operative recovery period of approximately 24 hrs, probes were perfused with artificial cerebrospinal fluid (ACSF, composition in mM: NaCl, 125; KCl, 2.5; MgCl_2_, 1.18 and CaCl_2_, 1.26) at a rate of 0.8 μl/min using a Harvard Apparatus infusion pump (mod. 22). After an initial 60 min equilibration period, dialysate samples were collected every 30 min for and 2.5 hrs to establish baseline release of glutamate and GABA in sham and SNI rats. Groups of rats received tetrodotoxin (TTX, 1 μM), or calcium-free ACSF, by reverse microdialysis to assess the synaptic nature of glutamate and GABA in mPFC cortex dialysates. On completion of experiments, rats were anaesthetised with pentobarbital and their brains perfused-fixed via the left cardiac ventricle with heparinised paraformaldehyde saline (4%). Brains were dissected out and fixed in a 10% formaldehyde solution for 2 days. Each brain was cut in 40 micron thick slices and observed under a light microscope to identify the probe location. Dialysates were analysed for amino acid content using a high-performance liquid chromatography (HPLC) method. The system comprised a Varian ternary pump (mod. 9010), a C18 reverse-phase column, a refrigerated autoinjector (mod. 9100), a fluorimetric detector (mod. PS363). Dialysates were pre-column derivatised with o-pthaldialdehyde (10 microliter dialysate + 10 microliter o-pthaldialdehyde) and amino acid conjugates resolved using a gradient separation. The detection limit of GABA and glutamate in 10 microliter samples was approximately 0.5-1 and 2-3 pmol, respectively. The mobile phase consisted of two components: (A) 0.1 M sodium acetate buffer (pH 6.95), 25% tetrahydrofuran and 10% methanol and (B) 100% methanol; gradient composition was determined with a Dell PC installed with Varian Star gradient management software, and the mobile phase flow rate was maintained at 1.0 ml/min. Data were collected by a Dell Corporation PC system 310 interfaced with Varian Star 6.2 control data and acquisition software. The mean dialysate concentration of amino acids in the first five samples represents the basal release in the two different groups of rats.

### RNA extraction and RT-PCR

Total RNA was extracted from homogenized mPFC using an RNA Tri-Reagent (Molecular Research Center Inc., Cincinnati, OH) according to the manufacturer's protocol. The extracted RNA was subjected to *DNase *I treatment at 37°C for 30 min. The total RNA concentration was determined by UV spectrophotometer. The mRNA levels of the genes under analysis were measured by RT-PCR amplification, as previously reported [59]. RT minus controls were carried out in order to check potential genomic DNA contamination. These RT minus controls were performed without using the reverse transcriptase enzyme in the reaction mix. Sequences for the mouse mRNAs from GeneBank (DNASTAR INC., Madison, WI) were used to design primer pairs for RT-PCRs (OLIGO 4.05 software, National Biosciences Inc., Plymouth, MN). Each RT-PCR was repeated at least four times so as to achieve optimal reproducibility data. A semi-quantitative analysis of mRNA levels was carried out using the "Gel Doc 2000 UV System" (Bio-Rad, Hercules, CA). The measured mRNA levels were normalised with respect to β-actin chosen as housekeeping gene. The β-actin gene expression values were expressed as arbitrary units ± SE. Amplification of genes of interest and β-actin were performed simultaneously.

### Western Blotting

For the protein extraction, the mPFC was minced into small pieces with a blender, then was suspended in lysis buffer (4% SDS, 20% glycerol, 10% 2-mercaptoethanol, 0.004% blue-bromophenol, Tris-HCl, pH 6.8, containing 6 M urea, 50 μM Na_3_VO_4_, 50 μM PMSF (Sigma Chemical Co., St. Louis, MO). The total protein concentration was determined using the method described by Bradford [60]. Each sample was loaded, electrophoresed in a 12% polyacrylamide gel and electroblotted onto a nitrocellulose membrane. Primary antibodies were used to detect TRPV1 and FAAH according to the manufacturer's instruction at 1:500 dilution (Santa Cruz; USA). Immunoreactive signals were detected with a horseradish peroxidase-conjugated secondary antibody and reacted with an ECL system (Amersham Pharmacia, Uppsala, Sweden). Protein levels were normalized with respect to the signal obtained with anti-beta-actin monoclonal antibodies (Sigma Chemical Co., St. Louis, MO, 1:1000 dilution).

### Immunohistochemistry

Under pentobarbital anaesthesia, animals were perfused transcardially with saline solution (0.9% NaCl) and 4% paraformaldheyde fixative. The brain was taken out and kept in the fixative for 24 h at 4°C. The tissue was kept in 30% sucrose in PBS and frozen in cryostat embedding medium (Bio-Optica, Milano, Italy). Serial 15 μm sections of the brain were cut using a cryostat and thaw-mounted onto glass slides. After washing in PBS, non-specific antibody binding was inhibited by incubation for 30 min in blocking solution (1% BSA, 0.2% powdered skim milk, 0.3% Triton-X 100 in PBS). Primary antibodies were diluted in PBS blocking buffer and slides were incubated overnight at 4°C in primary antibodies to goat polyclonal TRPV1 (1:100, Santa Cruz; USA) or goat polyclonal FAAH (1:100, Santa Cruz; USA). Fluorescent-labelled secondary antibodies (1:500; Alexa Fluor 488, Molecular Probe, Invitrogen, Carlsbad, CA) specific to the IgG specie used as a primary antibody were used to locate the specific antigens in each section. Sections were counterstained with bisbenzimide (Hoechst 33258, Hoechst, Frankfurt, Germany) and mounted with Vectashield mounting medium (Vector Laboratories, Burlingame, CA). Fluorescently labelled sections were viewed with a fluorescence microscope (Leica, Wetzlar, Germany) to locate the cells and identify the area of the brain.

### Analysis of endocannabinoid levels

In order to perform the endocannabinoid analysis, a different cohort of rats was used. Decapitation was performed and brains were rapidly removed and embedded in oxigenated ice-cold artificial cerebrospinal fluid. A PFC slice of 1.30-1.35 mm was cut throughout the PFC by using a vibrotome (Vibratome 1500, Warner Instruments, CT, USA) (interaural from +1.9 mm to +0.7 mm) [61]. The obtained slice of tissue containing the mPFC was then further dissected under optical microscope for microsurgery to isolate the PL-IL cortex (M650, Wild Heerbrugg, Switzerland) to be homogenized accordingly to our protocol. In brief, tissues were homogenized in 5 volumes of chloroform/methanol/Tris HCl 50 mM (2:1:1) containing 20 pmol of d^8^-AEA and d^5^-2-AG. Deuterated standards were synthesized from commercially available deuterated arachidonic acid and ethanolamine or glycerol, as described, respectively, in Devane et al. [62] and Bisogno et al. [63]. Homogenates were centrifuged at 13,000 × *g *for 16 min (4°C), the aqueous phase plus debris was collected and extracted again twice with 1 volume of chloroform. The organic phases from the 3 extractions were pooled and the organic solvents evaporated in a rotating evaporator. Lyophilized extracts were re-suspended in chloroform/methanol 99:1 by volumes. The solutions were then purified by open bed chromatography on silica as described in Bisogno et al. [63]. Fractions eluted with chloroform/methanol 9:1 by volume (containing AEA and 2-AG) were collected, the excess solvent was evaporated with a rotating evaporator, and aliquots were analysed by isotope dilution-liquid chromatography/atmospheric pressure chemical ionization/mass spectrometry (LC-APCI-MS) carried out under conditions described previously [64] and allowing the separation of the four compounds. Mass spectrometric (MS) detection was carried out in the selected ion monitoring mode using m/z values of 356 and 348 (molecular ions +1 for deuterated and undeuterated AEA) and 384.35 and 379.35 (molecular ions +1 for deuterated and undeuterated 2-AG). The area ratios between the signals of the deuterated and undeuterated compounds varied linearly with varying amounts of undeuterated compounds (30 fmol-100 pmol). AEA and 2-AG levels in unknown samples were therefore calculated on the basis of their area ratios with the internal deuterated standard signal areas. For 2-AG, the areas of the peaks corresponding to 1(3)-and 2-isomers were added together. The amounts of endocannabinoids were expressed as pmol/g or nmol/g of wet tissue weight.

### Drugs

*N*-arachidonoyl-serotonin (AA-5-HT) was synthesized in V. Di Marzo's laboratory as previously described [65]. 5'-Iodoresiniferatoxin (I-RTX), TTX and AM251 were purchased from Tocris Bioscience (Bristol, UK). 3'-carbamoylbiphenyl-3yl-cyclohexylcarbamate (URB597) was purchased from Cayman Chemical Co. (Germany). All drugs were dissolved in 0.5% DMSO in ACSF.

### Statistics

Microdialysis, behavioural and electrophysiology data are represented as means ± SE and statistical analysis of these data were performed by two way ANOVA for repeated measured followed by the Student-Newman-Keul for multiple comparisons to determine statistical significance between different treated groups of rats. For biomolecular analysis, protein quantification and immunohistochemistry the Student-Newman-Keuls and the Tukey tests have been used, respectively.

## Results

### Characterization of electrical stimulation-evoked responses of mPFC neurons

Single-unit extracellular recording in anesthetized rats was made from individual neurons in the prelimbic or infralimbic part of the mPFC (Figure 1B). Action potential duration (540 ± 20 μsec peak-to-valley) and firing rate (1.1 ± 0.5 spikes/s) from recorded neurons were consistent with presumed pyramidal cells rather than fast-spiking interneurons, the latter having a higher baseline firing rate (>10 Hz) and narrower spike waveform (< 300 microsec) [66,22,34].

### Electrophysiological properties of BLA-mPFC neurons in sham and SNI rats

We first investigated the proportion of mPFC neurons (n = 3-5 *per *rat) with ongoing activity that responded with inhibition [BLA→mPFC(-)], or excitation, [BLA→mPFC(+)]. In these studies, we first isolated mPFC neurons and thereafter stimulated the BLA at 0.5 Hz using an initial stimulation current of 700 μA. Whenever a neuron that was responsive to BLA stimulation was encountered, the BLA was stimulated with 100-200 pulses to determine whether the neuron responded with inhibition or excitation. The same procedure has been used for four groups of rats: 1) sham rats treated for 7 days with vehicle (sham/veh); 2) sham rats treated for 7 days with AA-5-HT (5 mg/kg, i.p.) (sham/AA-5-HT); 3) SNI rats treated for 7 days with vehicle (SNI/veh) and 4) SNI rats treated for 7 days with AA-5-HT (5 mg/kg, i.p.). In the control group (sham/veh), 80% of encountered neurons (n = 32) displayed an inhibition of spontaneous activity after BLA stimulation or mechanical stimulation, with the remaining proportion of cells showing an excitatory **response (n = 8)**. In SNI/veh rats, 70% of neurons (n = 28) displayed excitation after BLA or mechanical stimulation, while 30% of cells (n = 12) showed an inhibitory response. The 7 day period of repeated treatment with AA-5-HT (5 mg/kg, i.p.) in SNI altered the proportion between BLA→mPFC(-) and BLA→mPFC(+) neurons. Indeed 67.8% of BLA→mPFC(-) (n = 27) and 32.2% of BLA→mPFC(+) neurons (n = 13) were encountered.

### BLA→mPFC(-)

In BLA→mPFC(-) neurons the spontaneous firing rate was 1.1 ± 0.2 spikes/sec, the onset of BLA-evoked inhibition was 83.3 ± 7 ms and the duration of the inhibition was 463 ± 23 ms in sham/veh rats (2A, D and E). Treatment with AA-5-HT (5 mg/kg, i.p.) did not affect either the firing rate (1.12 ± 0.3 spikes/sec), the duration of the inhibition or the onset of inhibition of BLA→mPFC(-) neurons in the shams (not shown).

SNI/veh rats showed an increased firing rate (2.2 ± 0.5 spikes/sec) of BLA→mPFC(-) neurons. The onset of BLA-evoked inhibition was significantly (P < 0.05) reduced (32 ± 7.5 ms) in BLA→mPFC(-) neurons of this group of rats although no statistically significant changes were observed in the duration of the inhibition (455 ± 15 ms) (Figure 2B, D and 2E).

Treatment with AA-5-HT (5 mg/kg i.p.) for 7 days in SNI rats (SNI/AA-5-HT) decreased firing rate (1.2 ± 0.5 spikes/sec), caused a significant increase in the onset (175 ± 5 ms) of BLA-evoked inhibition and a significant (P < 0.05) decrease in the duration (250 ± 15 ms) (Figure 2C, D and 2E).

### BLA→mPFC(+) neurons

BLA→mPFC(+) neurons had a firing rate of 0.5 ± 0.2 spikes/sec in sham/veh group. The onset, the frequency and the duration of excitation were 47 ± 2.12 ms, 6.7 ± 0.8 spikes/sec and 380 ± 13.3 ms, respectively (3A, D, E and F). Sham rats treated for 7 days with AA-5-HT (5 mg/kg i.p.) did not show changes in the onset, the frequency and the duration of excitation (not shown) with respect to sham/veh. Rats which underwent SNI (SNI/veh,) showed a firing rate of 0.8 ± 0.2 spikes/sec. The onset of BLA-evoked excitation was significantly (P < 0.05) reduced (26.4 ± 3.4 ms) in this group of rats. The duration and the frequency of evoked excitation of BLA→mPFC(+) increased significantly (672.5 ± 13.43 ms and 10.3 ± 1.16 spikes/sec, respectively) in this group of rats (SNI/veh) (Figure 3B, D, E and 3F). Treatment with AA-5-HT (5 mg/kg i.p.) for 7 days in SNI rats caused a significant increase in the onset (46.8 ± 7.8 ms) and a significant reduction in the duration and the frequency of evoked excitation of BLA→mPFC(+) neurons (375 ± 16.37 ms and 7.7 ± 0.31 spikes/sec, respectively) (Figure 3C, D, E and 3F).

### Mechanical stimulation-evoked responses of BLA-mPFC neurons in sham or SNI rats

This cell population, previously identified by BLA electrical stimulation as BLA→mPFC(-) neurons, responded accordingly to noxious mechanical stimuli with an inhibition. The onset and duration of the mechanical stimulation-evoked inhibition was 93.7 ± 4.7 and 480 ± 23 ms, respectively (Figure 4A, D, E) in sham/veh rats. Treatment with AA-5-HT (5 mg/kg) did not affect either the duration or the onset of mechanical stimulation-evoked inhibition in the shams (sham/AA-5-HT). In the SNI/veh group of rats the onset of mechanical stimulation-evoked inhibition and its duration (60 ± 10 and 385 ± 9.5 ms, respectively), were significantly reduced (Figure 4B, D and 4E). Treatment with AA-5-HT (5 mg/kg i.p.) for 7 days in SNI rats caused a significant increase in the onset (210 ± 5.7 ms) and in the duration (550 ± 10 ms) of the mechanical stimulation-evoked inhibition (Figure 4C, D and 4E).

### Mechanical stimulation-evoked responses of mPFC BLA→mPFC(+)neurons

This cell population, preliminarily identified by BLA electrical stimulation as BLA→mPFC(+) neurons, responded accordingly to noxious mechanical stimuli with an excitatory response. The onset, the frequency and the duration of excitation of these mPFC neurons were 97 ± 8.5 ms, 400 ± 15 ms and 7.4 ± 1.2 spikes/sec, respectively in the sham/veh (Figure 5A, D, E and 5F) group of rats. Treatment with AA-5-HT (5 mg/kg, i.p.) did not affect either duration, onset and frequency of mechanical stimulation-evoked excitation in the shams (sham/AA-5-HT) (not shown).

SNI/veh rats showed a significantly reduced onset (65 ± 12.2 ms) and an increased duration and frequency of mechanical stimulation-evoked excitation (600 ± 10 ms and 12 ± 1.1 spikes/sec, respectively) (Figure.5B, D, E, F). Treatment for 7 days with AA-5-HT (5 mg/kg i.p.) caused a significant reduction in the duration (280 ± 12.2 ms) and frequency (6.2 ± 2.2 spikes/sec) of excitation in SNI/veh rats, whereas no change was observed in the onset (70 ± 2.8 ms) of mechanical stimulation-evoked excitation (Figure 5C, D, E and 5F).

### Microdialysis

**Figure 6:**
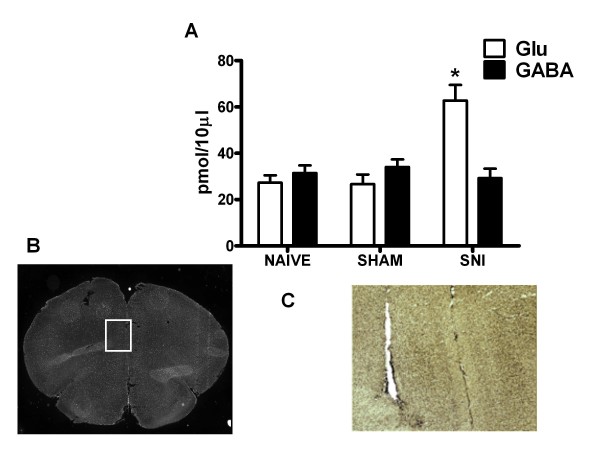
**"A" shows the release of glutamate and GABA in naïve, sham or SNI rats 7 days after injury**. The values of extracellular GABA and glutamate in the mPFC were expressed as pmol in 10 μl of perfusate. * indicate significant difference vs sham rats. Each point represents the mean ± S.E.M of 7-8 animals per group. P values < 0.05 were considered statistically significant. "B" shows a panoramic picture of the pre-frontal cortex, the square indicates the pre/infra-limbic area. "C" shows a high magnification of the microdialysis probe location for aminoacid collection within the the pre/infra-limbic cortex. Coronal brain slices containing the sites of implantation of the microdialysis probes were obtained after the experiment and processed for histological analysis.

The values of extracellular level of glutamate in PL/IL cortex were measured in pmol in 10 μl (pmol/10 μl). In vitro recovery of the microdialysis probe for glutamate was 22 ± 5%. The mean basal value for glutamate within the mPFC was 30.5 ± 6.2 pmol/10 μl. In sham rats (n = 7), the extracellular glutamate level in the mPFC did not change (27.3 ± 5.5 pmol/10 μl). Instead, the extracellular glutamate level increased significantly (62.8 ± 8 pmol/10 μl) (n = 8, P < 0.05) in SNI rats (Figure 6A). In vitro recovery of the microdialysis probe for GABA was 21 ± 4%. The mean basal values (not corrected for probe recovery) of extracellular GABA level in the mPFC were 32.5 ± 5.7 pmol/10 μl. The mPFC extracellular GABA was unchanged in sham and SNI rats as compared to the naives (Figure 6A).

### TRPV1 and FAAH expression in sham and neuropathic rats

**Figure 7:**
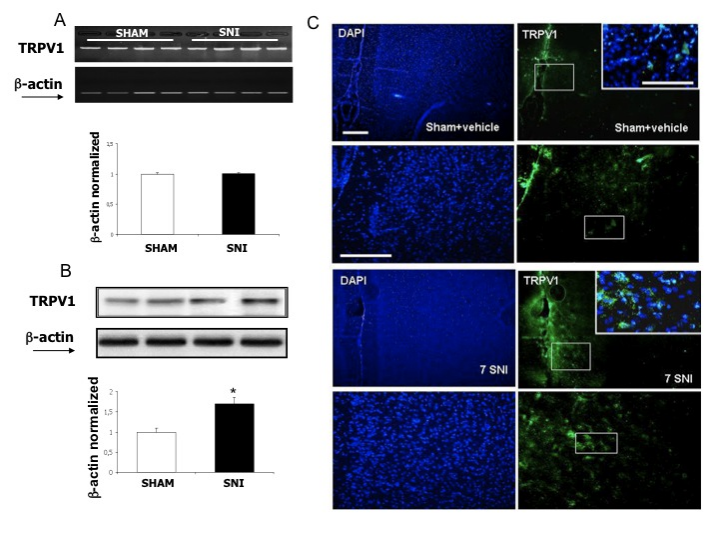
**TRPV1 mRNA and protein levels in the pre/infra-limbic cortex of sham and SNI rats**. "A" shows the unchanged TRPV1 mRNA levels normalized vs β-actin in neuropathic vs sham rats. "B" shows the enhanced TRPV1 protein levels normalized vs β-actin in neuropathic vs sham rats. "C" shows the increased TRPV1 staining in the layer II-III of the rat pre/infra-limbic cortex. Data are represented as a mean ± SEM n = 3 rats per group. P < 0.05 was considered statistically significant. ANOVA, post hoc Tukey. Scale bars = 100 μm.

We have observed that both targets of the AA-5-HT: the TRPV1 channel and FAAH were up-regulated in SNI as compared to the sham rats in the PL/IL cortex. In particular, we found that TRPV1 upregulation only occurred at the protein level, while mRNA levels did not increase significantly in neuropathic animals (Figure 7A, B, C). Conversely, FAAH mRNA levels were up-regulated, as well as the protein expression in the layer II-III of PL/IL cortex in neuropathic SNI animals, also indicating a possible change in the endocannabinoid turnover (Figuer 8A, B, C). Immunohistochemical data were obtained from the sham and SNI rats without any brain lesion.

**Figure 8:**
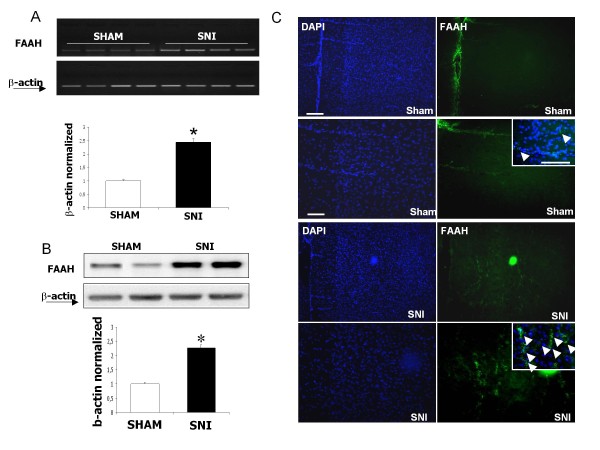
**FAAH mRNA and protein levels in the pre/infra-limbic cortex of sham and SNI rats**. "A" shows the enhanced FAAH mRNA levels normalized vs β-actin in neuropathic vs sham rats. "B" shows the enhanced FAAH protein levels normalized vs β-actin in neuropathic vs sham rats. "C" shows the increased FAAH staining in the layer II-III of the rat pre/infra-limbic cortex. Data are represented as a mean ± SEM n = 3 rats per group. P < 0.05 was considered statistically significant. ANOVA, post hoc Tukey. Scale bars = 100 μm.

### Intra-cortex microinjections of AA-5-HT, AM251, I-RTX or URB597 transiently inhibited allodynia in SNI rats

**Figure 9:**
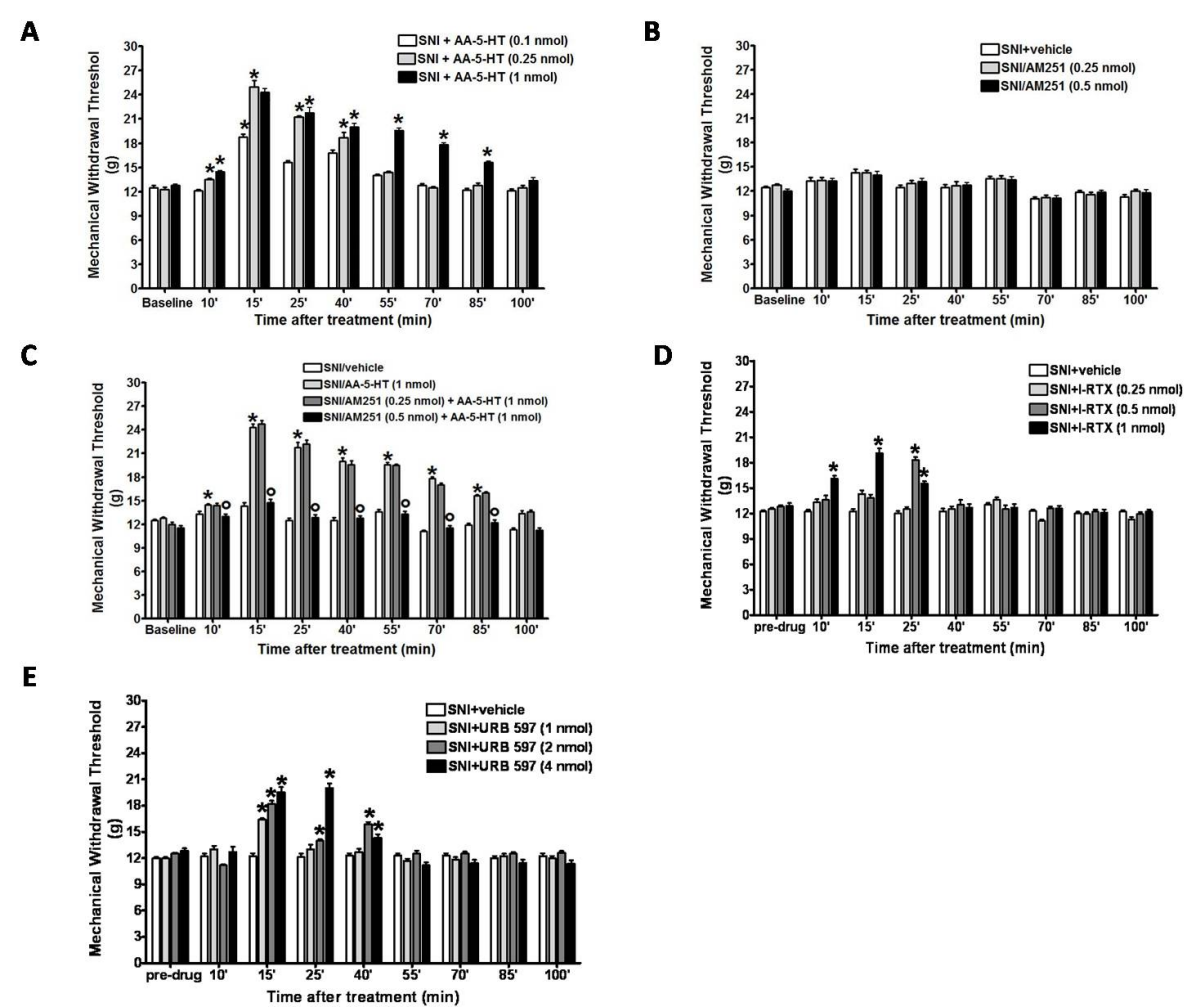
**Effects of a single injection of vehicle, AA-5-HT (0.1-0.25-1 nmol) (A), AM251 (0.25-0.5 nmol) (B), AM251+AA-5-HT (C), I-RTX (0.25-0.5-1 nmol) (D) or URB 597 (1-2-4 nmol) (E) on mechanical withdrawal threshold (mean ± S.E.M.) in spared nerve injury (SNI) rats**. Each point represents the mean ± S.E.M. of 8-10 rats per group. *p < 0.05 *vs *SNI/veh.

SNI of the sciatic nerve resulted in a significant decrease in mechanical withdrawal threshold in the ipsilateral side of rats, though not on the contralateral sides (12.5 ± 0.6 g) 7 days after surgery (Figure 9A,B and 9C). A single microinjection of AA-5-HT (0.1-0.25-1 nmol) (at day 7 after surgery) into the PL/IL cortex decreased mechanical allodynia in a dose-dependent manner and it was apparent up to 85 min after microinjection (Figuer 9A). This effect was antagonized by the co-injection with AM251 (0.5 nmol), a CB1 selective antagonist, while the same dose of AM251 per se did not exert any significant effect (Figure 9B, C). Conversely, a single microinjection of I-RTX (0.25-0.5-1 nmol) or URB597 (1-2-4 nmol) (at day 7 after surgery) both proved to be less effective in decreasing mechanical allodynia, as the effect lasted no longer than 10-40 min after injection (Figure 9D, E respectively) in SNI rats.

### Endocannabinoid and endovanilloid levels are altered in the PL/IL cortex of SNI rats

SNI was accompanied by a slight but statistically significant decrease in anandamide levels (from 27.2 ± 0.9 to 23.4 ± 0.4 pmol/g wet tissue weight, P < 0.05) in the PL/IL cortex, whereas the levels of 2-AG were slightly increased (from 2.1 ± 0.3 to 2.5 ± 0.2 nmol/g wet tissue weight, P < 0.05) (means ± SEM, N = 5).

## Discussion

Most studies on pain-related synaptic plasticity have focused on long-term changes at the peripheral and spinal dorsal horn neurons [67-74]. Pain-related synaptic reorganization in cortical areas, including the mPFC, anterior cingulate cortex (ACC), insular cortex [17,53,75-78] and BLA [34], and its contribution to pain processing or to the emotional-affective aspects of pain [30], has been less investigated. More recently however, some contrasting data have emerged on the phenotypic changes of mPFC pyramidal neurons which may depend on the pain models used. For instance, a large increase in the NMDA/AMPA ratio of the synaptic currents in layers II-III of PL/IL neurons, together with specific dendritic spine proliferation has been found in the SNI of the sciatic nerve model [16], whereas a massive mPFC neural deactivation and depression were recently observed in the arthritic pain model [34].

In the present study, by using integrative methods, we demonstrate that BLA→mPFC(-) inhibitory and BLA→mPFC(+) excitatory neurons, which concurrently respond to the hind-paw pressoceptive stimuli, show phenotypic changes in SNI-induced mono-neuropathy in the rat, suggesting that the mPFC may undergo profound reorganization related to chronic pain. Consistent with data by Metz et al. [16], the current study shows that SNI can shift the balance of excitatory and inhibitory responses in the BLA→mPFC pathway, resulting in a net increase in the excitatory influence that the BLA exerts over the PL/IL neuron population of the mPFC [30,79,80]. Indeed, whilst in sham rats we found that the majority (about 80%) of the pyramidal neurons belongs to the inhibitory BLA→mPFC(-)subtype, with the remaining part being excitatory neurons of the BLA→mPFC(+) subtype, in SNI rats such a ratio was nearly the opposite. One of the main reasons for the strong presence of inhibitory cells in physiological conditions might be that GABAergic interneurons are mainly interfaced between BLA-driven excitatory input and the PL/IL pyramidal neurons of the mPFC [81,82]. Moreover, cortex GABAergic interneurons show a very strong responsiveness to excitatory inputs because of the faster AMPA-mediated cationic gating in such interneurons than in excitatory pyramidal cells under basal conditions [83,84]. Intriguingly, in this study a critical difference was detected between neuropathic and sham rats in the excitatory BLA→mPFC(+) neurons. A decrease in the onset, enhancement of frequency and a longer duration of evoked excitation following ipsilateral BLA electrical stimulation was observed in this study. However, our electrophysiological parameters, in particular as it regards the duration of evoked excitation are extremely longer than those found by Floresco and Tse [53] and Laviolette and Grace [22]. Differences among the anaesthetics used during the electrophysiological procedures could be responsible of the discrepant results observed. In particular, Floresco and Tse and Laviolette and Grace [53,22] have used a high dose of urethane (1.5 g/kg, i.p. instead of the more conventional 1.2 g/kg, i.p.) for maintaining anaesthesia. Urethane has a complex multi-target (still poorly known) mechanism of action such as a non-selective positive modulation of GABAA and GlyR receptors and a depression of the NMDA and AMPA glutamate receptors. Indeed, these anaesthetics very rarely allows a complete recovery from the anaesthesia. Collectively, the high dose and the multi-target mechanism of action of it may justify the decreased duration of the evoked excitation in the pyramidal neurons observed by Floresco and Tse and Laviolette and Grace [53,22]. In BLA→mPFC(-) neurons, the onset of the inhibition decreased in SNI rats, suggesting that inhibitory neurotransmission might be down-regulated in this cortex area during a pathological painful condition. Indeed, in vivo microdialysis experiments performed here in awake rats showed that the extracellular levels of glutamate increased in the contralateral mPFC cortex of SNI rats, with no measurable change in GABA levels under the same experimental conditions. Overall, these data suggest an SNI-induced imbalance between the excitatory and inhibitory amino-acidergic neurotransmissions, resulting in the increased excitability of the layers II/III pyramidal cells of the mPFC cortex. Consistently, mechanical noxious stimulation applied to the contralateral paw evoked excitatory or inhibitory responses in the cell populations previously identified by BLA electrical stimulation. The application of noxious stimuli to the contralateral paw of SNI rats resulted in a decreased onset of burst or pause for the excitatory or inhibitory cells, respectively. As far as the other analyzed functional parameters are concerned, an increased frequency and duration of excitation were observed following paw mechanical stimulation. Collectively, these in vivo physiological data support recent ex vivo findings indicating that mPFC pyramidal neurons undergo profound morpho-functional reorganization associated with SNI-induced neuropathic pain, supporting the possibility of major involvement of the layers II/III of PL/IL cortex in the patho-physiological processes associated with the unpleasant or the affective component of pain [16]. Although the details of the pain-related BLA-driven changes justifying the enhanced excitatory synaptic activity on PL/IL pyramidal cells are yet to be determined, the pharmacological, electrophysiological, biochemical and morphological data from the current and previous studies seem consistent with a polysynaptic pathway.

Indeed, even if the glutamatergic BLA projection to GABAergic mPFC inter

 could explain the BLA-driven inhibitory responses in about 80% of the PL/IL pyramidal neurons in normal conditions [81,82], it remains to be determined why the excitatory/inhibitory cell populations ratio shifted dramatically in favour of the excitatory cells in the SNI pain model. One possibility could be a strengthened direct connection between excitatory glutamatergic BLA impinging on pyramidal neurons of PL/IL cortex rather than on the inhibitory interneurons [85-87], caused by SNI-induced proliferation of mPFC pyramidal neuron dendrites [16]. Alternatively, another possible explanation might be that the increased SNI-induced endovanilloid tone, i.e. the over-expression of the TRPV1 channel, may lead to the increased release of glutamate in the PL/IL cortex, since TRPV1 activation is well known to be coupled to enhanced glutamate release in the brain [88]. Indirect evidence supporting this possibility comes from the present finding that SNI was also accompanied by increased FAAH expression and the subsequent decrease of the levels of the endocannabinoid/endovanilloid anandamide in the PL/IL cortex, as well as by an increase in the levels of the endocannabinoid 2-AG. These latter events might represent an adaptive mechanism aiming at providing a negative feed-back control on the putative TRPV1-mediated stimulation of glutamate release, since anandamide is an endogenous activator of TRPV1, and 2-AG, which is inactive at TRPV1, may instead inhibit glutamate release by acting as a retrograde signal at pre-synaptic CB_1 _receptors. Alternatively, the stimulation by 2-AG of presynaptic CB1 receptors on GABAergic fibers might contribute to reduce inhibitory signalling in the PL/IL cortex, even though we did not observe here any reduction in extracellular GABA levels in microdyalisates from this brain region of SNI rats. Mechanistic studies in mice with SNI are under way in order to investigate the role of the endocannabinoid and endovanilloid system in the enhanced excitatory vs. inhibitory signalling observed here in SNI rats.

It is worth noting that a relatively short temporal window (7 days after SNI) was sufficient to produce the observed morphological, neurochemical and functional changes. These data are consistent with previous evidence of increased NMDA receptor subunit NR2B in the cingulate cortex of mice with persistent pain [89], as well as with the reported synaptic proliferation on basal dendrites of pyramidal neurons in the mPFC cortex in SNI rats [16]. Such a morpho-functional reorganization at the neuron basal dendrite level would indicate a specific long-lasting neuro-adaptive process aiming at straightening the intra-cortical circuits, more than the extra-cortical ones, in a way that such an increased local spine density would wrongly integrate inputs converging in this area.

A further consideration concerns the evidence that the current data from 7-day treated SNI rats do not seem to be consistent with data obtained in patients suffering from neuropathic pain, in whom atrophy of the limbic prefrontal cortex was reported [10]. However, as previously suggested by Metz et al. [16], it is possible that this neuropathic pain model and the short period of observation (which in fact requires a longer interval of time in order to induce apoptosis or glutamate-mediated excitotoxicity) did not allow us to note any apparent neurodegeneration. Indeed there is no reason to exclude the possibility that following a longer period of over-excitation, increased spine number and NMDA receptor currents may lead to increased glutamatergic excitotoxicity and apoptosis.

Based on the over-expressed endocannabinoid/endovanilloid biomarkers and elevated glutamate levels, we decided to perform in SNI rats a repeated daily systemic treatment with AA-5-HT, which we had previously demonstrated to produce anti-allodynic and anti-hyperalgesic effects in the chronic constriction injury (CCI) neuropathic pain model via both indirect activation/desensitization of TRPV1 and activation of cannabinoid CB_1 _receptors, following elevation of AEA and 2-AG levels, and direct TRPV1 receptor antagonism [49]. Indeed, AA-5-HT exerts its analgesic effect in three ways: i) antagonism of TRPV1 receptors involved in thermal hyperalgesia, ii) desensitization of TRPV1-expressing nociceptors involved in mechanical allodynia and iii) indirect agonism at cannabinoid CB_1 _receptors in the CCI model of neuropathic pain [49]. Here we report that AA-5-HT treatment was able to prevent mechanical allodynia and modulate both inhibitory and excitatory transmission in the BLA-mPFC pathway. Intriguingly, the relative efficacy of the effects of intra-PL/IL cortex microinjections of selective vanilloid (I-RTX) or FAAH (URB597) inhibitors or of the "hybrid" compound, AA-5-HT, underline the importance of concentrating two activities in one single molecule to significantly reduce allodynia and further validate the critical role played by TRPV1 and FAAH in specific mPFC sub-regions involved in pain modulation. Several previous studies have reported that the PL/IL cortex, which corresponds to the dorsal-lateral prefrontal cortex in humans, plays a crucial role in pain processing [10,89-93], and emerging imaging studies show that this brain region is involved in pain inhibition in humans [93]

Furthermore, since the treatment with AA-5-HT led to restore normal neuronal activity in the BLA-mPFC pathway, these data support our hypothesis that the over-expressed TRPV1 channel, which seems to be mainly present in glutamatergic neurons, is one of the mechanisms that in SNI rats activate pathways (likely calcium-dependent) associated with cell plasticity [94,95]. In agreement with the TRPV1-induced neural plasticity described previously [42,96-98], it is possible that functional re-organization mediated by glutamate/endovanilloid and GABA/endocannabinoid signalling can also occur following TRPV1 channel over-stimulation and the consequent increased release of glutamate in the PL-IL cortex of SNI rats in both identified populations of mPFC neurons.

In conclusion, this study shows that in pressoceptive responding populations of mPFC neurons, the previously described BLA→mPFC(-) and BLA→mPFC(+) neurons [53], may undergo profound reorganization related to neuropathic pain. The present findings indicate that a relatively short period of SNI-induced neuropathy (7 days) may be sufficient to up-regulate the endovanilloid/endocannabinoid machinery in the PL/IL cortex, implying that disruptions in the mPFC endovanilloid/endocannabinoid system might impair behaviours mediated by the BLA-mPFC circuits. Since similar alterations have been shown in corresponding neural circuitries in chronic pain subjects, it may be conceivable to speculate that the changes we have observed here could be a contributing factor to emotional and cognitive disturbances associated with chronic pain disorders. Local or systemic pharmacological manipulation of the TRPV1 channel and the enzyme FAAH with the hybrid drug AA-5-HT proves to inhibit allodynia/hyperalgesia and normalize the imbalance between excitatory and inhibitory responses in the mPFC neurons. As such, psychopharmacological therapies designed to normalize endovanilloid/endocannabinoid transmission in the mPFC glutamatergic terminals may prove useful in alleviating the symptoms and central sequelae of neuropathic pain syndromes.

## Competing interests

The authors declare that they have no competing interests.

## Authors' contributions

VdN has conceived and conceptualized the study, DV, LG and SB have performed treatments, electrophysiology and behaviour experiments, LL has carried out immunohistochemistry, GB and DS have performed molecular biology, MdC and IM have performed microdialysis, SM and EP have written the manuscript. FP has performed analysis of endocannabinoid level. VdM has participated in the design of the study and provided AA-5-HT. FR has contributed to the drafting of the study. All authors have read and approved the final manuscript.
